# Supplementary material for: Minimally invasive surgery and neurophysiological monitoring for brainstem hemorrhage: Advancing predictive models with qEEG and TCD
Source: PLoS One. 2025 Oct 21;20(10):e0323850. doi: 10.1371/journal.pone.0323850 (PMC12539704; doi:10.1371/journal.pone.0323850)
Supplement: Attachment — Submitted filename: Response to Reviewers.docx [file pone.0323850.s002.docx]

**Response to Editor**

“PONE-D-25-17756 Minimally Invasive Surgery and Neurophysiological Monitoring for Brainstem Hemorrhage: Advancing Predictive Models with qEEG and TCD

ACADEMIC EDITOR REQUESTS:

Major corrections:

1. Please describe the approaches taken to the brainstem and how often each was utilized. That is, which entry zone was used? If surgery was done under CT, how were you able to define the anatomy to specify the entry zone? Also, please give a better description of both the conventional and stereotactic approaches: a reader of your manuscript should be able to recreate what you've done after reading a scientific manuscript.

Response: We have added a new subsection “Entry Zones for Brainstem Hemorrhage” and” Table 1” in Methods that details the candidate entry zones and our selection algorithm.

2. Please provide dosing for all drugs mentioned (e.g. urokinase). Additionally, since Urokinase is being used in an off-label fashion, this needs to be specified in the manuscript and justified using prior publications describing that use.

Respouse: We have added dosing information for urokinase. “The use of urokinase for intracavitary clot lysis is off-label, and prior clinical studies have supported its reasonableness: it accelerates the resolution of intracranial hematomas and, in selected cohorts, when standardized protocols are followed, improves clinical outcomes without excess symptomatic hemorrhage. Drawing on representative trials and systematic reviews in spontaneous intracerebral hemorrhage and intraventricular hemorrhage that used similar dosing ranges, we instilled 20,000 IU diluted in 2 mL of preservative-free normal saline into the hematoma cavity, followed by a 1–2 mL saline flush; the catheter was clamped for 30–60 minutes and then reopened for drainage. Dosing was repeated every 12 hours for up to 3–5 days, with a maximum cumulative dose of 120,000–200,000 IU, and was stopped earlier if CT showed adequate evacuation or if rebleeding was suspected.”

3. Statements such as " Importantly, no patients experienced complications such as rebleeding, further demonstrating the safety and efficacy of this surgical approach" or "The findings of this study demonstrate that stereotactic aspiration surgery provides significant advantages over conventional therapy in improving functional outcomes for patients with brainstem hemorrhage" are perhaps not justified points to make, given the data provided. The "safety" or "superiority" of this surgery should not commented on for several reasons: 1) the n is too small to make such claims, 2) not enough data/description is given to validate whether the treatment groups were properly balanced to allow for comparison: was only one surgeon involved in stereotactic approach vs one surgeon for conventional approach? Were multiple surgeons involved? What was the decision process to use one technique vs the other? Were patients randomized to treatment? If not, what decision making went into choosing one option vs another? Not enough detail is given. The authors are encouraged to describe the techniques and the outcomes without making values-based statements regarding safety or efficacy.

Response：We thank the reviewer for this important point.

- Original:“Importantly, no patients experienced complications such as rebleeding, further demonstrating the safety and efficacy of this surgical approach.”Revised to:“We did not observe symptomatic rebleeding. However, given the small sample and nonrandomized design, no inferences about safety can be drawn.”
- Original:“The findings of this study demonstrate that stereotactic aspiration surgery provides significant advantages over conventional therapy in improving functional outcomes for patients with brainstem hemorrhage.”Revised to:“Our findings are descriptive and hypothesis generating. While some outcomes appeared numerically favorable in the stereotactic group, the study was not designed or powered to establish superiority over conventional therapy.”

4. Minor corrections: Line 464 "analyses. he use of postoperative qEEG in the" The authors likely mean "The use of" Please rigorously check the manuscript for typos and grammar errors before considering resubmitting.

Response：Thank you for the reminder; we have made the corresponding changes.

5. If applicable, we recommend that you deposit your laboratory protocols in protocols.io to enhance the reproducibility of your results. Protocols.io assigns your protocol its own identifier (DOI) so that it can be cited independently in the future.

Response：Thank you for the suggestion. As this study depositing on protocols.io is not applicable. We have provided methodological details and parameters in the Methods.

**Journal Requirements:**

1. Please ensure that your manuscript meets PLOS ONE's style requirements, including those for file naming.

Response: The corresponding changes have been made.

2．Thank you for stating in your Funding Statement: “This work was supported by the Key Scientific and Technological Project of Zhejiang Province, including the Quzhou City Guided Project (Grant No. 2021022 and Grant No. 2018086), as well as the Zhejiang Medical Association Clinical Research Fund Project (Grant No. 2022ZTC-A111).” Please provide an amended statement that declares all the funding or sources of support (whether external or internal to your organization) received during this study, as detailed online in our guide for authors at <http://journals.plos.org/plosone/s/submit-now>. Please also include the statement “There was no additional external funding received for this study.” in your updated Funding Statement. Please include your amended Funding Statement within your cover letter. We will change the online submission form on your behalf.

Response: The corresponding changes have been made.

3. Thank you for stating the following financial disclosure:Please state what role the funders took in the study.

Response: The funders had the following involvement with the study: study design, decision to publish, and preparation of the manuscript.

4. We note that you have indicated that there are restrictions to data sharing for this study. For studies involving human research participant data or other sensitive data, we encourage authors to share de-identified or anonymized data. However, when data cannot be publicly shared for ethical reasons, we allow authors to make their data sets available upon request. For information on unacceptable data access restrictions, please see <http://journals.plos.org/plosone/s/data-availability#loc-unacceptable-data-access-restrictions>.

Respouse: All relevant data are available from Figshare at <https://doi.org/10.6084/m9.figshare.30018478>.

5. Please include your tables as part of your main manuscript and remove the individual files. Please note that supplementary tables (should remain/ be uploaded) as separate "supporting information" files".

Response: The corresponding changes have been made.

6. We note that Figure 2 includes an image of a patient in the study.

Respouse: We have obtained written informed consent for publication using the PLOS consent form. We have revised the Methods and Ethics statement to explicitly state this and added a note to the Figure 2 legend. The signed consent form has been securely filed in the patient’s case notes and will not be submitted.

**Review Comments**

Reviewer #1: The sample size is small (n=25); a power analysis or justification is missing.TCD parameters improved post-surgery but showed no correlation with mRS; explanation in discussion is limited.Lack of external validation or multicenter data limits generalizability.The timeframe between monitoring and outcome assessment (90 days) is appropriate but not clearly discussed in terms of neurological recovery stages.

Response: Given the rarity of primary brainstem hemorrhage, our sample consists of consecutive cases accrued within a predefined period (n=25). TCD indices capture acute cerebrovascular hemodynamics, whereas mRS reflects global disability influenced by multiple factors such as baseline hematoma volume and location, initial severity, secondary complications, and rehabilitation. Additionally, mRS may exhibit floor/ceiling effects in severe brainstem injury, and any relationship may be non-linear or time-dependent. We agree. The single-center design limits generalizability. In our study, 90‑day mRS was pre‑specified as the primary outcome to align with common practice in stroke research and to facilitate comparability across studies. A detailed staging framework of neurological recovery would require longitudinal clinical scales and serial assessments that were beyond the scope and data structure of the present work. To avoid speculation, we kept the discussion concise. No changes were made in response to this comment, but we would be happy to elaborate if the Editor deems it necessary.

Reviewer #2: nicely written article. It highlight an area where not much progress has been made yet. the results indicates more work necessary to improve existing treatment paradigms for primary brainstem hemorrhage.

Response: We thank Reviewer #2 for the positive feedback and encouragement. We agree that this is an area with limited progress, and our findings underscore the need for continued efforts to refine treatment paradigms for primary brainstem hemorrhage.
