# Supplementary material for: Minimally invasive surgery and neurophysiological monitoring for brainstem hemorrhage: Advancing predictive models with qEEG and TCD
Source: PLoS One. 2025 Oct 21;20(10):e0323850. doi: 10.1371/journal.pone.0323850 (PMC12539704; doi:10.1371/journal.pone.0323850)
Supplement: Attachment — Submitted filename: renamed_28ca7.docx [file pone.0323850.s003.docx]

Academic Editor comments:

1. It appears that patients receiving "conventional therapy" received only supportive care and no surgery. If that is correct, please strongly consider referring to "conventional therapy" as "non-operative management" to avoid confusion. Also, in the discussion, the "conventional group" is called the "non-surgical group." For the sake of simplicity, please pick one name for each group and use it consistently throughout.

**Respond**：Thank you very much for your detailed and important suggestions regarding the terminology. We have made the corresponding changes in the manuscript and highlighted them in yellow.

2. Lines 348-350. Please either remove this or change it to say something like "There was no significant difference in mortality between the treatment groups." The 30 day survival rate showed no significant difference, so you should not present this as a change. You have defined statistical significance as p<0.05, so any change that does not meet this definition is not a true change and should not be elaborated on as such. Any other nonsignificant differences should be treated similarly.

**Respond:** Based on the reviewers' comments and standard practices for reporting statistical significance, we have made the following changes to this paragraph: "There was no significant difference in the 30-day survival rate between the stereotactic surgery group and the non-surgical group (p = 0.36). However, a significant difference was observed in the modified Rankin Scale (mRS) scores at 90 days (p = 0.01). In the stereotactic surgery group, 68.75% of patients achieved favorable outcomes (mRS score 0–3), whereas only 11.11% of patients in the non-surgical group reached this outcome. Conversely, poor outcomes (mRS score 4–6) were more prevalent in the non-surgical group (88.89%) compared to the stereotactic surgery group (31.25%)." and highlighted the changes in yellow.

3. Because this is retrospective, because you did not randomize treatment, because your sample size is quite small, please do not write that one treatment is superior to another. Lines 439-441 still read that "stereotactic aspiration provides significant advantages over conventional therapy" and your study is not robust enough to make this claim.

**Respond：**Based on the comment and the limitations of our study design, we have revised the original sentence—“The findings of this study demonstrate that stereotactic aspiration surgery provides significant advantages over non-operative management in improving functional outcomes for patients with brainstem hemorrhage.”—to: “Our retrospective findings indicate a possible association between stereotactic aspiration surgery and improved functional outcomes relative to non-operative management, but confirmation in larger randomized or prospective studies is needed.” and highlighted the changes in yellow.

4. Again in the conclusion, reference is made to "the superiority" of a multi-modal approach. The study is not designed to address superiority, so I would remove that or significantly change the language. The next line reads that there are "clinical benefits" to stereotactic aspiration. At best, because this is a non-randomized retrospective study, you could say that the surgical approach with aspiration correlates with a lower modified Rankin scale. The line after that reads "improves prognostic accuracy" and "enhances functional outcomes." Again, please remove this language or significantly change it. The next line reads that "the findings support the incorporation . . . better long-term recovery in patients with brainstem hemorrhage. Again, please remove this language or significantly change it. In the manuscript, there are multiple occurrences of this type of language (claims not supported by the data presented). Please change these.

**Respond:** We agree with the reviewers’ comments and have revised the deterministic and causal terms “superiority,” “clinical benefits,” “improves/enhances,” and “support the incorporation.” The conclusion has been updated to:“This study highlights the prognostic value of qEEG and aEEG parameters in patients with brainstem hemorrhage and suggests that a multi modal predictive approach may offer enhanced prognostic utility compared with single parameter assessments. Additionally, our data indicate that stereotactic aspiration surgery is associated with reduced hematoma volume, alleviation of brainstem compression, and mitigation of secondary injury mechanisms. The integration of neurophysiological monitoring with surgical intervention shows promise for optimizing patient management strategies. While these observations require validation in prospective controlled studies, they provide a foundation for further exploration of personalized treatment protocols for brainstem hemorrhage.” and highlighted the changes in yellow.

5. In the manuscript, there are multiple occurrences of this type of language (claims not supported by the data presented). Please change these.

**Respond:** We agree with the reviewer’s comments. In the Abstract, the Background section was revised from “Stereotactic hematoma aspiration surgery offers an effective treatment, and postoperative brainstem function monitoring is crucial for optimizing patient outcomes.” to “Stereotactic hematoma aspiration surgery has been explored as a treatment option, and postoperative brainstem function monitoring is considered important for patient management.”

In the Abstract’s Results section, “Compared to non-surgical group, stereotactic surgery showed no significant differences in baseline characteristics but resulted in higher favorable outcomes at 90 days” was revised to “Stereotactic surgery was associated with higher rates of favorable outcomes at 90 days compared with the non-surgical group.”

In the Abstract’s Conclusion section, “This study demonstrates the prognostic value of qEEG and highlights the benefits of combining neurophysiological monitoring with stereotactic aspiration surgery. The integration of these tools improves prognosis and functional outcomes, supporting their routine use in clinical practice.” was revised to “This study suggests the prognostic value of qEEG and explores the utility of combining neurophysiological monitoring with stereotactic aspiration surgery. The integration of these tools may assist in prognostic assessment for PBSH patients; however, validation in larger prospective studies is required before clinical adoption.”

In the Discussion section of the main text, “Given the high level of trauma and technical complexity associated with craniotomy, stereotactic aspiration has emerged as a promising minimally invasive alternative for the treatment of intracerebral hemorrhage” was revised to “Given the high level of trauma and technical complexity associated with craniotomy, stereotactic aspiration has been proposed as a minimally invasive technique for intracerebral hemorrhage.”

Additionally, “Unlike conventional surgical approaches, stereotactic aspiration avoids the use of traction, electrocautery, or tissue separation during the procedure, significantly reducing the risk of iatrogenic injury” was revised to “Stereotactic aspiration is performed without traction, electrocautery, or tissue separation, which has been proposed to reduce iatrogenic injury.”

“The positive correlation between hematoma volume and mRS scores aligns with previous findings, reaffirming its role as a critical determinant of poor neurological outcomes. Larger hematoma volumes impose greater pressure on vital brainstem structures, resulting in worse functional recovery. However, the moderate predictive performance of hematoma volume alone (AUC = 0.718) highlights its limitations in providing accurate prognostic information, necessitating the incorporation of additional neurophysiological markers.” was revised to “The positive correlation between hematoma volume and mRS scores is consistent with prior reports of an association with poorer neurological outcomes. Larger hematoma volumes, which can exert greater pressure on vital brainstem structures, were associated with worse functional recovery. Given the moderate predictive performance of hematoma volume alone (AUC = 0.718), adding neurophysiological markers may have value for prognostic assessment.” and highlighted the changes in yellow.
